# Reversal of Obesity-Related Spinal Epidural Lipomatosis after Metabolic and Bariatric Surgery: A Case Suggesting the Role of Ectopic Fat Reduction

**DOI:** 10.70352/scrj.cr.26-0178

**Published:** 2026-06-13

**Authors:** Yota Tanahashi, Akira Sasaki, Akira Umemura, Yusuke Suto, Takafumi Iwasaki, Akiko Kawakami, Toma Kawashima, Hiroyuki Nitta

**Affiliations:** Department of Surgery, School of Medicine, Iwate Medical University, Shiwa-gun, Iwate, Japan

**Keywords:** spinal epidural lipomatosis, metabolic and bariatric surgery, ectopic fat, obesity, sleeve gastrectomy

## Abstract

**INTRODUCTION:**

Spinal epidural lipomatosis (SEL) is a disorder characterized by excessive accumulation of epidural adipose tissue, resulting in spinal canal stenosis and neurological symptoms. In recent years, obesity-related SEL has been increasingly recognized as a manifestation of ectopic fat deposition associated with metabolic dysfunction and has been re-evaluated as a potentially reversible condition following weight reduction. However, reports describing radiological and clinical improvement of SEL after metabolic and bariatric surgery (MBS) remain limited, and this disease concept and therapeutic strategy have not yet gained sufficient attention in Japan. Herein, we report a case of SEL that improved following MBS.

**CASE PRESENTATION:**

A 49-year-old man with severe obesity (body weight 160 kg; BMI 48.3 kg/m^2^) presented with several obesity-related diseases. MRI revealed spinal canal stenosis due to SEL with accompanying neurological symptoms, which did not improve despite medical weight-loss therapy. Laparoscopic sleeve gastrectomy was performed as MBS. At 1 year postoperatively, body weight had decreased to 122.4 kg (BMI 36.9 kg/m^2^), corresponding to 23.5% total weight loss and 43.2% excess weight loss. The cross-sectional area of epidural fat (EF) decreased from 4.22 cm^2^ preoperatively to 2.25 cm^2^ postoperatively, while the dural sac (DS) area increased from 0.61 to 2.32 cm^2^. Accordingly, the EF-to-DS (EF/DS) ratio decreased from 6.92 to 0.97, and the Borré classification improved from Grade III to Grade I. Neurological symptoms were alleviated, and no spinal decompression surgery was required.

**CONCLUSIONS:**

MBS led to meaningful improvement in patient-oriented outcomes accompanied by radiological resolution of SEL. Obesity-related SEL may represent a reversible condition driven by ectopic fat accumulation, and MBS may serve as a therapeutic option in carefully selected patients.

## INTRODUCTION

Spinal epidural lipomatosis (SEL) is a condition characterized by excessive accumulation and proliferation of unencapsulated mature adipose tissue within the spinal epidural space, leading to spinal canal stenosis and neurological symptoms such as low back pain, leg pain, and intermittent claudication.^1,2)^ In cases accompanied by severe neurological impairment, surgical decompression, including laminectomy, is often required.^1)^

The most common etiological factors associated with SEL are obesity and exogenous steroid use.^1,2)^ In addition, endogenous hypercortisolism such as Cushing’s syndrome, hypothyroidism, and idiopathic cases have also been reported.^1,2)^

In recent years, obesity-related SEL has been increasingly recognized not merely as localized fat overgrowth but as a manifestation of ectopic fat deposition associated with metabolic dysfunction, and it has been re-evaluated as a potentially reversible condition following weight reduction.^3–5)^ In Japan, however, the therapeutic strategy of positioning obesity-related SEL as a reversible condition and prioritizing obesity treatment before surgical decompression has not yet been widely established.

Herein, we report a case of SEL in which neurological symptoms improved following metabolic and bariatric surgery (MBS) without the need for spinal decompression surgery.

## CASE PRESENTATION

A 49-year-old man suffering from left lower leg numbness and pain, accompanied by low back pain, visited the Department of Orthopedic Surgery for evaluation. At presentation, he required cane-assisted ambulation due to these symptoms. His body weight was 168.4 kg, corresponding to a BMI of 50.8 kg/m^2^. Lumbar MRI revealed spinal canal stenosis caused by excessive epidural fat (EF) accumulation, and he was diagnosed with SEL (**Fig. 1A**–**1C**). Axial imaging demonstrated the characteristic Y-sign. Quantitative assessment using Synapse Vincent (Fujifilm, Tokyo, Japan) demonstrated an EF area of 4.22 cm^2^ and a dural sac (DS) area of 0.61 cm^2^ (EF to DS [EF/DS] ratio 6.92). According to the classification by Borré et al.,^2)^ this case was Grade III. Quantitative MRI assessment was performed at the L4/L5 level, where EF compression and the Y-sign were most clearly demonstrated, allowing reproducible pre- and postoperative comparison.

**Fig. 1:**
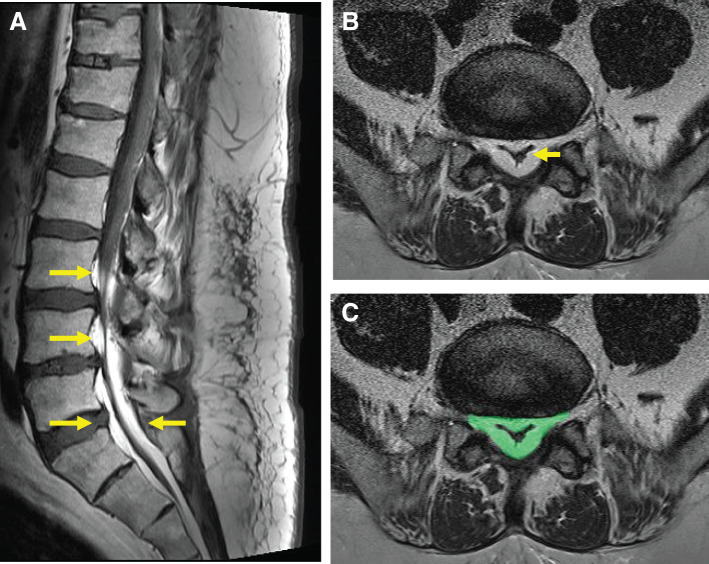
Preoperative lumbar MRI and quantitative analysis. (**A**) T1-weighted sagittal image showing marked narrowing of the DS from L3 to S1. Yellow arrows indicate excessive EF causing compression of the DS. (**B**) Axial T2-weighted image at the L4/L5 level demonstrating compression of the DS by hypertrophied EF, presenting the characteristic Y-sign. (**C**) Quantitative MRI analysis using Synapse Vincent (Fujifilm, Tokyo, Japan). At the same intervertebral level, the cross-sectional areas of EF (green) and the DS were measured. Preoperatively, the epidural fat area was 4.22 cm^2^ and the dural sac area was 0.61 cm^2^, yielding an EF/DS ratio of 6.92. DS, dural sac; EF, epidural fat; EF/DS, epidural fat-to-dural sac

Because obesity was considered a major contributing factor to SEL, weight reduction was recommended before decompression surgery. The patient was referred for evaluation of MBS. Assessment revealed hypertension, type 2 diabetes, obstructive sleep apnea, and metabolic dysfunction–associated steatohepatitis (MASH). Despite lifestyle intervention and pharmacotherapy, a total weight reduction of 8.4 kg was achieved; however, further weight loss plateaued, and no improvement in neurological symptoms was observed. Body weight immediately before surgery was 160.0 kg. Therefore, MBS was proposed for severe obesity and associated metabolic disorders, and laparoscopic sleeve gastrectomy was performed without complications. At 3 months postoperatively, body weight decreased to 137 kg, accompanied by improvement in low back pain. At 6 months postoperatively, body weight further decreased to 128.6 kg, and numbness in the left lower leg was alleviated, allowing the patient to ambulate without a cane. At 1 year postoperatively, body weight decreased to 122.4 kg (BMI 36.9 kg/m^2^), corresponding to 23.5% total weight loss and 43.2% excess weight loss. Various parameters of obesity-related diseases markedly improved at 1 year after surgery (**Table 1**).

**Table 1:** Changes in body weight and obesity-related metabolic parameters after MBS

Variables	Preoperative	Postoperative (1 year)
Body weight (kg)	160.0	122.4
BMI (kg/m^2^)	48.3	36.9
TWL (%)	—	23.5
EWL (%)	—	43.2
SFA (cm^2^)	636.4	421.1
VFA (cm^2^)	343.3	203.5
LV (mL)	1846.0	1374.0
SBP (mmHg)	143	106
DBP (mmHg)	91	65
TG (mg/dL)	80	77
TC (mg/dL)	156	120
LDL-C (mg/dL)	103	69
HDL-C (mg/dL)	35	29
IRI (μU/mL)	19.9	12.0
FG (mg/dL)	112	93
HbA1c (%)	6.3	5.7
HOMA-IR (no unit)	5.5	2.7
HOMA-β (no unit)	146.2	144
AST (U/L)	62	19
ALT (U/L)	70	20
PLT (×10^3^/µL)	201	189
FIB-4 index	1.80	1.27
APRI	0.77	0.29
MASLD fibrosis score	1.23	0.79
Fatty liver index	99.2	82.4

Changes in body weight, body composition indices, blood pressure, lipid metabolism, glucose metabolism, liver function, and fibrosis-related parameters before surgery and at 1 year postoperatively are shown.

ALT, alanine aminotransferase; APRI, aspartate aminotransferase-to-platelet ratio index; AST, aspartate aminotransferase; DBP, diastolic blood pressure; EWL, excess weight loss; FG, fasting glucose; FIB-4, fibrosis-4 index; HbA1c, hemoglobin A1c; HDL-C, high-density lipoprotein cholesterol; HOMA-β, homeostasis model assessment of β-cell function; HOMA-IR, homeostasis model assessment–insulin resistance; IRI, immunoreactive insulin; LDL-C, low-density lipoprotein cholesterol; LV, liver volume; MASLD, metabolic dysfunction–associated steatotic liver disease; MBS, metabolic and bariatric surgery; PLT, platelet count; SBP, systolic blood pressure; SFA, subcutaneous fat area; TC, total cholesterol; TG, triglyceride; TWL, total weight loss; VFA, visceral fat area

Follow-up MRI demonstrated marked reduction of EF (**Fig. 2A**–**2C**). The EF area decreased to 2.25 cm^2^ and the DS area increased to 2.32 cm^2^. The EF/DS ratio improved to 0.97, and the Borré classification improved to Grade I (**Table 2**). Neurological symptoms were further alleviated at 1 year. Health-related QOL assessed using the EQ-5D-5L questionnaire improved from 0.62 to 0.86. The patient has since maintained weight reduction without recurrence of obesity-related comorbidities and has not required spinal surgery.

**Fig. 2:**
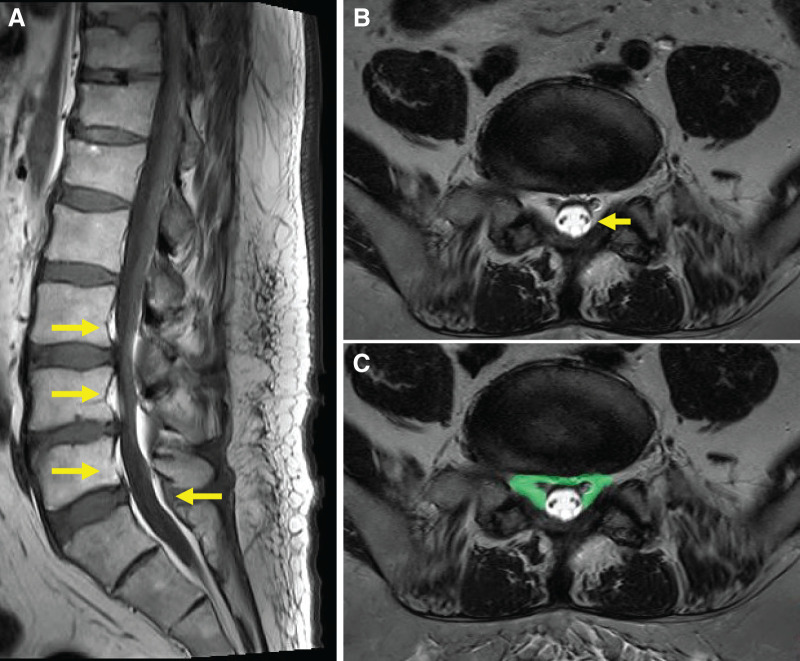
Lumbar MRI and quantitative analysis at 1 year after surgery. (**A**) T1-weighted sagittal image showing improvement of DS narrowing. Yellow arrows indicate the previously affected regions, demonstrating reduction of EF and alleviation of compression. (**B**) Axial T2-weighted image at the L4/L5 level demonstrating reduced compression by EF and resolution of the Y-sign. (**C**) Quantitative MRI analysis at the same intervertebral level revealed that the EF area decreased to 2.25 cm^2^, while the DS area increased to 2.32 cm^2^. The EF/DS ratio decreased to 0.97. DS, dural sac; EF, epidural fat; EF/DS, epidural fat-to-dural sac

**Table 2:** Quantitative MRI assessment of SEL before and after surgery

Variables	Preoperative	Postoperative (1 year)
EF area (cm^2^)	4.22	2.25
DS area (cm^2^)	0.61	2.32
EF/DS ratio	6.92	0.97
Borré grade	Grade III	Grade I

Axial MRI images at the same intervertebral level (L4/L5) were used to measure the cross-sectional areas of EF and the DS preoperatively and at 1 year postoperatively. Measurements were performed using Synapse Vincent (Fujifilm, Tokyo, Japan).

DS, dural sac; EF, epidural fat; EF/DS, epidural fat-to-dural sac; SEL, spinal epidural lipomatosis

## DISCUSSION

The improvement in neurological symptoms paralleled the postoperative weight loss process, suggesting a potential association between reduction in EF and clinical recovery. In the present case, MBS resulted not only in expected weight loss and metabolic improvement but also in radiological improvement of SEL and alleviation of neurological symptoms. Quantitative MRI analysis demonstrated a reduction in EF area and an increase in DS area, accompanied by a decrease in the EF/DS ratio and improvement in the Borré classification. These objective findings represent a key feature of this case.

SEL is characterized by excessive deposition of mature adipose tissue within the epidural space, leading to spinal canal stenosis and compression of neural elements, thereby causing neurological symptoms.^1,2)^ Standard management consists primarily of removal of underlying causes—such as steroid dose reduction—and conservative weight reduction strategies, while surgical decompression is typically reserved for patients with severe symptoms.^1)^ However, because SEL frequently develops in individuals with obesity or hypercortisolemic states, these patients often present with elevated perioperative risk, creating a clinical dilemma in which decompressive surgery itself may pose substantial procedural risk.

Although SEL is often regarded as a rare condition, epidemiological studies based on spinal MRI have demonstrated that it occurs at a measurable frequency.^3,4)^ Malone et al. reported a prevalence of 6.26% among patients with spinal stenosis, noting that all SEL cases were overweight and that 79% were obese.^3)^ Theyskens et al. similarly reported SEL findings in approximately 2.5% of patients undergoing spinal MRI.^4)^ Furthermore, a meta-analysis by Zhang et al. identified obesity as a significant risk factor for SEL.^5)^ These findings suggest that SEL may be present in a substantial proportion of patients with obesity.

In recent years, obesity has been increasingly redefined not merely as elevated BMI but as a systemic disease characterized by excess adiposity and organ dysfunction. In particular, ectopic fat deposition—defined as fat accumulation in organs and tissues not primarily designed for fat storage—has been implicated in metabolic abnormalities and organ dysfunction.^6)^ Ectopic fat depots such as epicardial fat, hepatic fat, pancreatic fat, and peripharyngeal fat have been associated with inflammation and functional impairment.^7–9)^ When fat accumulates within anatomically confined spaces, its mass effect itself may contribute to organ dysfunction.

In the present case, the reduction in EF can be interpreted within the framework of reversible ectopic fat deposition. Ectopic fat is known to decrease following weight reduction interventions,^7–9)^ and the observed improvement in SEL in this patient is consistent with this pathophysiological model. Recent studies have suggested that MBS induces changes in adipokine profiles, including decreased leptin levels and increased adiponectin levels, as well as improvements in systemic inflammation and lipid metabolism.^6–9)^ These changes may contribute to the preferential reduction of ectopic fat depots, including EF, although the precise mechanisms remain to be elucidated.

Rubino et al. proposed the concept of “clinical obesity,” defined as excess adiposity associated with organ dysfunction, distinguishing it from “preclinical obesity.”^10)^ The present patient had hypertension, type 2 diabetes, obstructive sleep apnea, and MASH, consistent with the phenotype of clinical obesity. MBS is positioned as a treatment for clinical obesity, and its impact on reducing ectopic fat deposition may have contributed to the improvement in SEL observed in this case.

While nonsurgical weight reduction is considered a therapeutic approach for SEL, the degree of improvement may vary among patients.^1)^ In the present case, substantial and sustained weight loss achieved through MBS may have contributed to the marked reduction of EF and improvement of neurological symptoms. In situations where avoidance of decompression surgery is desired, a more reliable and significant degree of weight reduction may be required, and MBS may represent a potential therapeutic option in selected patients.

Although several case reports have described improvement of SEL after MBS, quantitative radiological assessment combined with patient-oriented outcomes remains limited.^11,12)^ A summary of previously reported cases is presented in **Table 3**. These reports consistently demonstrate that substantial weight reduction following MBS is associated with improvement in both clinical symptoms and radiological findings. However, the number of reported cases remains limited, and further accumulation of evidence is required. At present, SEL is not specifically listed as an indication for MBS in the guidelines of the International Federation for the Surgery and Other Therapies for Obesity (IFSO) or the American Society for Metabolic and Bariatric Surgery (ASMBS).^13,14)^ However, given that obesity is a recognized risk factor for SEL and that weight reduction may lead to both radiological and clinical improvement, obesity-associated SEL may represent a condition warranting further investigation.

**Table 3:** Reported cases of SEL treated with MBS

Author (year)	Age/sex	Preoperative weight (kg)	Preoperative BMI (kg/m^2^)	Procedure	Postoperative weight	Follow-up (months)	Outcome
Valcarenghi et al. (2018)^11)^	48/M	131	43.0	Sleeve gastrectomy	95	12	Symptomatic and radiological improvement
Alsofyani et al. (2021)^12)^	55/M	120	—	Sleeve gastrectomy	89	6	Resolution of symptoms and MRI improvement
Present case	49/M	160	48.3	Sleeve gastrectomy	122.4	12	Marked clinical and radiological improvement

Previously reported cases are summarized, including preoperative body weight, BMI, surgical procedure, postoperative body weight, follow-up duration, and clinical and radiological outcomes. Although the number of reported cases remains limited, substantial weight reduction achieved after metabolic and bariatric surgery has been consistently associated with improvement in both symptoms and imaging findings.

MBS, metabolic and bariatric surgery; SEL, spinal epidural lipomatosis

Taken together, even in patients in whom surgical decompression would otherwise be considered based on symptom severity, MBS may contribute to disease modification in obesity-related SEL. In this case, MBS performed for severe obesity and obesity-related comorbidities resulted in reduction of EF, improvement of spinal canal stenosis, and alleviation of neurological symptoms.

In contrast, weight reduction achieved with preoperative medical management did not result in clinical improvement in this case, suggesting that a greater magnitude of weight loss may be necessary to achieve meaningful improvement in SEL. In the present case, a modest weight reduction of 8.4 kg achieved through preoperative medical management did not result in clinical improvement, whereas substantial weight loss of approximately 40 kg following MBS was associated with marked alleviation of neurological symptoms and radiological improvement.

This observation raises the possibility that a certain magnitude of weight reduction may be required to achieve meaningful improvement in obesity-related SEL. However, given that this report describes a single case, the existence of a specific threshold cannot be determined. Further studies with larger cohorts are needed to clarify whether a quantitative relationship exists between the degree of weight loss and improvement in SEL.

Quantitative MRI assessment enabled objective demonstration of reduced EF deposition and improvement of neural compression following a systemic fat-reducing intervention. From the perspective of reversible ectopic fat accumulation, MBS may represent a potential therapeutic strategy in selected patients with obesity-associated SEL. This report describes a single case, and a causal relationship between MBS and improvement of SEL cannot be definitively established. Because SEL extended across multiple lumbar levels, a single-level assessment may not fully represent the overall disease burden. The possibility that spontaneous changes or other unmeasured factors contributed to the observed improvement cannot be excluded. Furthermore, the long-term durability of the radiological and patient-oriented improvements remains uncertain. Accumulation of additional cases and prospective studies is required to clarify the therapeutic role of MBS in obesity-associated SEL.

## CONCLUSIONS

MBS resulted in meaningful patient-oriented improvement accompanied by radiological resolution of SEL. Obesity-related SEL may represent a reversible condition driven by ectopic fat accumulation, and MBS may serve as a potential therapeutic strategy in selected patients.

## DECLARATIONS

### Availability of data and materials

All relevant data supporting the findings of this study are included within the article.
